# Periprosthetic Infection of Transfibular Ankle Arthroplasties Managed with Implant Retention: Anatomical Limitations of Surgical Debridement

**DOI:** 10.3390/antibiotics14030215

**Published:** 2025-02-21

**Authors:** Pilar Hernández-Jiménez, Mikel Mancheño-Losa, María Ángeles Meléndez-Carmona, M Ángela Mellado-Romero, Patricia Brañas, Carlos Lumbreras-Bermejo, Jesús Enrique Vilá y Rico, Jaime Lora-Tamayo

**Affiliations:** 1 Department of Internal Medicine, Hospital Universitario “12 de Octubre”, Instituto de Investigación Sanitaria Hospital “12 de Octubre” (imas12), PC 28041 Madrid, Spain; mikel.mancheno@gmail.com (M.M.-L.); carlos.lumbreras@salud.madrid.org (C.L.-B.); sirsilverdelea@yahoo.com (J.L.-T.); 2 Department of Clinical Microbiology, Hospital Universitario “12 de Octubre”, Instituto de Investigación Sanitaria Hospital “12 de Octubre” (imas12), PC 28041 Madrid, Spain; marmelcar@gmail.com (M.Á.M.-C.); patriciamaria.branas@salud.madrid.org (P.B.); 3 Foot and Ankle Unit, Department of Orthopaedics and Traumatology Surgery, Hospital Universitario “12 de Octubre”, PC 28041 Madrid, Spain; mariaangela.mellado@salud.madrid.org (M.Á.M.-R.); vilayrico@gmail.com (J.E.V.y.R.); 4 Centro de Investigación Biomédica en Red de Enfermedades Infecciosas (CIBERINFEC), Instituto de Salud Carlos III (ISCIII), PC 28029 Madrid, Spain

**Keywords:** ankle arthroplasty, transfibular, prosthetic joint infections

## Abstract

**Background:** Prosthetic ankle infection is an infrequent and rarely explored prosthetic joint infection (PJI). In early infection, the debridement of implants inserted using the transfibular approach has certain peculiarities that pose a diagnostic and therapeutic challenge, the impact of which on infection prognosis is still unknown. **Methods:** This study prospectively collected all cases of transfibular prosthetic ankle infection at a tertiary hospital between 2014 and 2022, describing their demographic, clinical, microbiological, and management characteristics, along with the outcome over a long follow-up. This cohort was compared with a cohort of infected fibular plates without prostheses implanted in the same period of time. **Results:** Seven cases of ankle PJI were analysed, all of them implanted using a transfibular approach. They were all early prosthetic infections. The median age was 63 years (range 54–74) with a predominance of women (71.4%), three patients with diabetes (42.9%), and one patient with rheumatoid arthritis (14.3%). The aetiology was predominantly staphylococcal (4 [57.1%] methicillin-susceptible *S. aureus* and 1 [14.3%] *S. epidermidis*). All cases were managed with irrigation and debridement limited to the fibular plate, four of which failed (57%). By comparison, eleven cases of infected fibular plates without prostheses implanted were analysed. There were no differences in clinical, microbiological, or therapeutic management characteristics between the groups. Failure among infected fibular plates occurred in only two cases (18%). **Conclusions:** Debridement of infected transfibular ankle prostheses suggests a worse evolution than would be expected for other joint infections. This could be explained by the nature of the debridement, limited to the fibular component. Further detailed studies of the surgical possibilities in prosthetic ankle infections are necessary to improve the prognosis of these infections, given their impact on joint function.

## 1. Introduction

**Figure 1:**
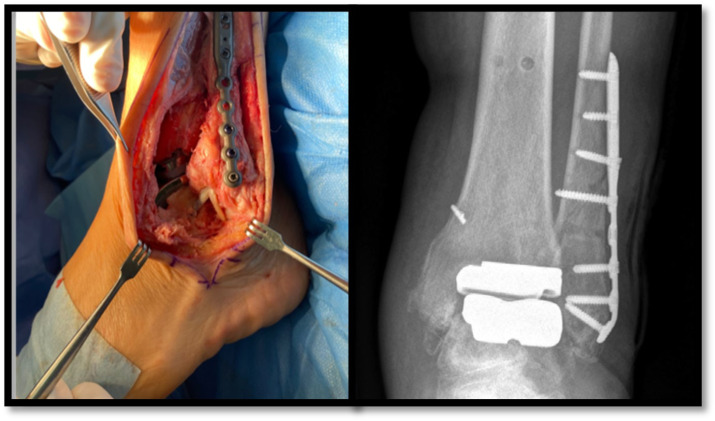
Transfibular approach in an ankle prosthetic implant. Surgical and radiological view. Adapted with permission from Ref. [2]. Copyright 2023, American College of Foot and Ankle Surgeons.

Ankle arthroplasty is increasingly being used for the treatment of ankle osteoarthritis [1]. In recent years, the transfibular approach has been shown to have functional advantages over the classic anterior incision, despite being more technically demanding and requiring the implantation of osteosynthesis material in the fibula (Figure 1) [2].

Prosthetic joint infection (PJI) after ankle arthroplasty is less common compared to hip or knee PJI, but the incidence is higher (2–10%) [3,4]. The indications for the treatment of ankle PJI are the same as for knee and hip PJI [5]. However, there is limited experience in the appropriate management of ankle PJI in implants inserted using the transfibular approach, especially when debridement with implant retention is indicated. Re-opening the fibular route is controversial in this scenario because of the subsequent risk of instability and the difficulties of fibular bone resynthesis, which would jeopardise the mechanical goals of the prosthesis. However, this can lead to suboptimal debridement of the prosthetic components and thus a theoretically higher probability of failure. By contrast, in ankle prostheses placed through the anterior approach, an iterative incision leads directly to the prosthetic compartment, allowing for the proper debridement of the prosthetic components.

In our centre, starting in 2014, a progressive switch from the anterior approach to the lateral prosthesis was made, based on the best-published results of this new technique [2,6,7]. The aim of this study was to analyse our experience in the management of prosthetic ankle infections (PAI) by focusing on the peculiarities of transfibular approach with special attention to cases managed with irrigation and debridement limited to the fibular plate. We analysed and compared the results with a cohort of infected fibular plates without ankle prostheses with the aim of delimiting the involvement of the prosthetic component in the process of infection.

## 2. Results

Among a total of 291 episodes of prosthetic joint infection (PJI) identified in our centre, 10 happened in ankle arthroplasties (3.4%) (7 transfibular and 3 anterior ankle prostheses). The total number of ankle prostheses implanted during the described period was 84 in the transfibular approach, resulting in an overall infection incidence of 8.3% (7/84). Among the seven cases of transfibular PAI, arthroplasty was indicated in four patients (57%) with post-traumatic injury, two (29%) with arthrosis, and one (14%) with an inflammatory chronic disease. Two patients (29%) had undergone a prior lateral approach for their original orthopaedic disease. The median age was 64 years (range 54–74), and five (71%) were women. Diabetes was the most common baseline condition [three cases (43%)]. There was only one case of rheumatoid arthritis with corticosteroid and immunosuppressant therapy, with no cases of cancer or other immunosuppressants. Three patients (43%) were active smokers. Other patients’ baseline conditions are resumed in Table 1.

During the period 2018–2022, there were eleven cases of infection related to osteosynthesis material in fibular fractures with a median age was 56 years (range 39–86), six (55%) being women. Comorbidities were distributed in 27% (three) diabetes and 18% (two) rheumatoid arthritis and chronic pneumopathy each. Eight patients (73%) had an early infection and four (27%) had a delayed infection [8]. Only three patients were smokers (27%). The main aspects of these cases are summarised in Table 2. 

Infection was classified as early post-surgical in all the cases of PJI. Median time from initial surgery to the diagnosis of infection was 40 days (range 11–82). All the patients presented with wound dehiscence, three of them with local inflammatory signs and pain (43%), and fever in two cases (28.6%). Radiological loosening and sinus tract were absent in all cases. Aetiology was polymicrobial in three cases (43%). The main bacteria involved were *Staphylococcus aureus* (four cases, 57%, all of them methicillin-susceptible), followed by *Enterobacter cloacae* (two cases, 29%) and *S. epidermidis* (one case, 14%). X-rays of the ankle showed prostheses stability in all cases. No CTs or MRIs were performed prior to the surgical treatment.

**Figure 2:**
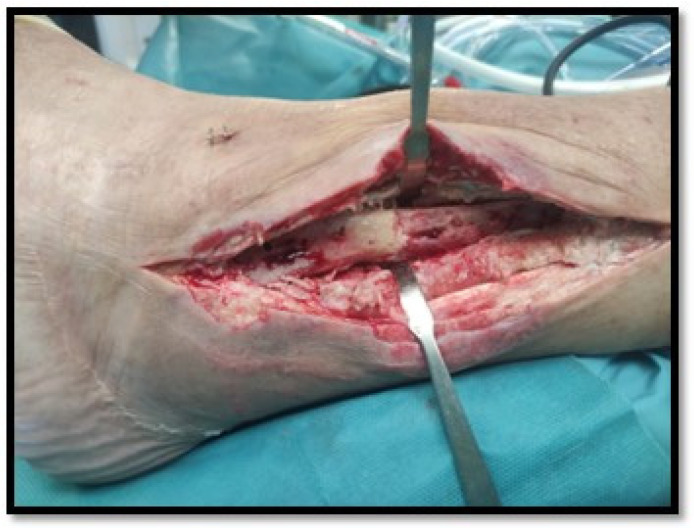
Transfibular surgical approach in one of the patients. Osteosynthesis material removal.

The main aspects of therapeutic management are summarised in Table 3. All seven patients were managed with irrigation and debridement (I&D) of the fibular plate by an iterative surgical transfibular approach, with two cases requiring an anterior compartment dissection conducted through the same surgical approach. The osteosynthesis fibular plate could be removed in three cases (43%) (Figure 2). To prevent joint instability, in none of these seven cases was the joint space approached or the prosthetic components debrided, nor the polyethylene liner exchanged. Four cases required flap wound coverage and two cases needed vacuum therapy. In four cases, wound recovery was unfavourable requiring further wound closure therapies.

The empirical treatment protocol encompassed the parenteral administration of β-lactam antibiotics exhibiting antipseudomonal activity, along with either vancomycin or daptomycin. Subsequent to a 3–7-day interval, therapeutic adjustments were made based on microbiologic findings, with a transition to oral administration over a total duration of 8 weeks.

Three patients (43%) were considered cured and unassisted walking was achieved in all three. The median follow-up time was 0.9, 7 and 7.3 years, respectively. The other four patients (57%) presented with failure after a median of 220 days, in the range of 60–582. Salvage therapy consisted of a second I&D of the fibular plate with arthroscopy associated in one case (25%), a removal of the fibular osteosynthesis material in one case (25%), and two-stage revision in two cases (50%), involving the placement of a cement spacer loaded with vancomycin and vancomycin plus clindamycin in one case each. All four cases were again approached via transfibular. Median follow-up in this group was of 3.5 years (range 2.6–6.4). As per functional outcome, at last follow-up two patients were able to walk without support (Table 2).

A comparison between patients with a favourable and unfavourable outcome revealed no differences in demographic or clinical characteristics except for a non-significant higher frequency of diabetes mellitus in failed cases (3/4 versus 0/3; *p* = 0.14). Polymicrobial infection was also more frequent in failed cases (3/4 vs. 0/3, *p* = 0.14). A trend towards a longer delay from the onset of symptoms to surgery was observed among failed (median 59 days, range 56–363) versus cured cases (median 21.5 days, range 5–38) (*p* = 0.06). This delay may have contributed to the evolution of acute infections into chronic infections in some cases. We did not observe a higher rate of failure among patients with a need for soft tissue coverage (*p* = 0.714).

The eleven patients with a fibular plate infection (no prosthetic) had similar basic characteristics to patients with PJI. The main microbiological aetiology was methicillin-susceptible *S. aureus* (73%). Seven cases were treated with the removal of osteosynthesis material (64%) and four cases with I&D (36%). Infection recurred in only two cases (18%, one I&D and one two-stage revision), both of which were salvaged by orthopaedic hardware removal. A non-statistically significant higher rate of infection recurrence was observed among patients with transfibular ankle PJI than in cases of fibular plate-associated infection (57% vs. 18%, *p* = 0.087).

## 3. Discussion

Ankle PJI is an emerging disease that has increased in recent years with advances in orthopaedic techniques but remains infrequent. Despite being a less frequent arthroplasty than knee or hip prostheses, the incidence of PAI is higher, probably due to its anatomical nature. To our knowledge, this is the first study focusing on the management of infected transfibular ankle prostheses. While previous studies have reported the incidence of infection in anterior devices, as well as the differences between that technique and the transfibular approach regarding functional outcomes, none of these has addressed the technical problems of the surgical management of PAI, especially for transfibular arthroplasties [3,4,9].

The treatment of PAI is often based on guidelines from other PJI locations. While I&D has proven to be as effective as in the hip or knee joints for the classic anterior approach, our results show a considerable failure rate for infected transfibular ankle arthroplasties (57%). This rate is higher than that reported in other series of infection in prostheses implanted by an anterior approach; Mazzoti et al. reported a rate of 48%, while Lachman et al. reported a rate of 54%, which may have been influenced by the incidence of methicillin-resistant *S. aureus* [5,10,11]. The patients in our series had similar baseline conditions and microbiological aetiologies to those reported in other series [11,12]. We hypothesise that suboptimal debridement of the joint compartment in patients with a transfibular ankle prosthesis may contribute to the poor outcomes observed in some cases. This hypothesis is supported by the comparison between transfibular ankle prostheses and infected peroneal plates, which suggests that the presence of intra-articular orthopaedic material could be a key factor responsible for the higher failure rate observed in the former group.

Indeed, a meticulous joint debridement and cleaning of orthopaedic material is at the heart of the surgical treatment. It is essential to remove as much necrotic material, debris, and purulence as possible, as well as bacterial biofilm [13]. This, however, may be problematic for a transfibular ankle prosthesis: on the one hand, reopening the transfibular route (refracturing and re-repairing the fibula) can lead to joint instability; on the other, not accessing the joint compartment and restricting lavage to the peroneal plate may lead to suboptimal debridement and ultimately failure.

Given the difficulties of reusing the transfibular approach, the question of how to perform debridement in these patients remains open. While a new anterior incision of the ankle could lead to problems of skin ischaemia [9], the possibility of an arthroscopic lavage could perhaps be explored. In other prosthetic joints, arthroscopy carries poorer results in I&D compared with open debridement, among other reasons, because the exchange of removable components is not possible [14,15]. However, some authors report successful outcomes with arthroscopic debridement of knee prostheses using a supplementary posterior port [16,17]. Analogically, in such a specific and complex scenario as transfibular PAI, the combination of a peroneal debridement plus an arthroscopic lavage could be an acceptable approach to preserve joint stability and ameliorate the thoroughness of the debridement. Alternatively, a less invasive re-synthesis of the fibula (e.g., revision of the fibula osteotomy with K-wires or screws intramedullary to avoid hardware over the fibula) could also be explored to provide the necessary capacity for deep joint surgical exploration while preserving the stability of the joint.

Our study has some limitations. Firstly, it was conducted at a single centre, and the number of cases was too small to obtain conclusive results. Secondly, the joint cavity was not explored, which could cast doubt on the involvement of the joint in the infection, except when it recurred. Lastly, the orthopaedic function assessment scale employed during follow-up was a basic tool that did not discriminate fine degrees of functionality or specific ranges of joint mobility. Nevertheless, this clinical problem has not previously been specifically addressed, and its strengths must also be noted. It is a long, prospective study conducted over 10 years at a renowned orthopaedic centre with extensive experience in osteoarticular infections.

In conclusion, the optimal surgical management of acute infections of transfibular ankle prostheses is challenging due to the risk of loss of joint stability. Our results show a high rate of failure of treatment when debridement is limited to the fibular component, suggesting that complementary surgical manoeuvres are needed. Larger multicentre studies are needed to confirm these results and consolidate collective experience with this technique.

## 4. Materials and Methods

This is an observational, prospective, single-centre study including all cases of transfibular PAI between 2014 and 2022 treated in a tertiary hospital. The centre is a 1300-bed acute-care teaching hospital with an Orthopaedic Unit that serves as a referral centre for a patient population of 550,000 inhabitants. Ethical approval for this study was obtained from the Hospital Research Ethics Committee (ref. 19/145).

Prostheses implanted via transfibular were TM AnkleR (ZimmerBiomet, Warsaw, IN, USA). The demographic characteristics of the identified cases were collected, including sex, age, comorbidities, immunosuppression status, type of prosthesis (primary or revision), infection characteristics (clinical, radiological, microbiological), and type of treatment administered (surgical and antibiotic therapy).

Cases of infection were defined according to the European Bone Joint Infection Society (EBJIS) [18]. All microbiological results were obtained exclusively from intraoperative tissue samples or joint fluid aspirations. The type of infection was classified according to the modified Tsukayama classification, in which early post-surgical infection was defined as infection presenting with symptoms in the first 90 days after implantation [19,20].

The treatment followed individualised and multidisciplinary recommendations of the Spanish national guidelines for PJI (SEIMC), with irrigation and debridement being indicated for acute infections (i.e., early post-surgical and haematogenous) when appropriate, or removal of the prosthesis for chronic infections, with one- or two-stage revision or arthrodesis at the discretion of the responsible orthopaedic surgeon [21]. All previous surgical incisions were used whenever possible, and in cases with significant soft-tissue loss, coverage techniques were employed (e.g., flaps or vacuum therapy). Functional rehabilitation of transfibular prostheses during the postoperative period took longer compared to the anterior approach, with mobility and partial carrying beginning 3 and 6 weeks after the surgery, respectively. Patients were followed up during hospitalisation and later in the outpatient clinic.

The primary endpoint was the failure of the first surgical and medical treatment used, defined as infection-related death, persistence or recurrence of infection, and/or the need for salvage therapy during follow-up, including suppressive antimicrobial therapy.

Since I&D of these cases was limited to the fibular plate, we compared the results with a cohort of patients with peroneal fracture-related infection, also involving the placement of a peroneal plate but without a joint prosthesis.

Data analysis was performed using the STATA 15.1 statistical software package. The Wilcoxon rank-sum test was used to compare continuous variables with non-normal distributions, while Fisher’s exact test was used for categorical variables. Confidence intervals were set at 95%, with a significance level of *p* < 0.05 for all tests. This study adhered to the STROBE guidelines for cohort studies [22]. The reporting checklist may be found in Table S1.

## Figures and Tables

**Table 1:** Demographic characteristics of the episodes of transfibular ankle prosthetic joint infection.

Case Number	Age (Years)	Diabetes Mellitus	Body Mass Index (kg/cm^2^)	Smoking	Aetiology of Arthropathy	Prior Lateral Approach
1501	54	No	NA	NA	Post-traumatic	Yes
1540	58	No	26	Ex-smoker	Post-traumatic	NA
3002	64	No	24	Smoker	Post-traumatic	No
1929	74	Yes	27	Smoker	Post-traumatic	Yes
2010	61	No	26	Smoker	Arthrosis	No
1819	64	Yes	44	Non-smoker	Arthrosis	No
3001	65	Yes	33	NA	Inflammatory	NA

NA: not available.

**Table 2:** Characteristics, management, and outcome of patients with fibular plate infection without prosthetic component.

Case Number	Age (Years) and Sex	Comorbidities	Days from Implant to Infection	Surgical Therapy	Additional Techniques	Microbiological Aetiology	Antibiotics *	Follow-Up/Time to Failure (Months)	Outcome	Salvage Therapy	Functional Outcome **	Radiology at Last Follow-Up
F1812	72 M	DM, BMI 25.2	42	OMR	No	*S. aureus* MS	Cefuroxime	5	Cure	-	1	Consolidated
F1822	40 M	BMI 30	28	Partial OMR	No	*S. aureus* MS	Levofloxacin	21	Cure	-	0	Consolidated
F2002	58 W	DM, BMI 28.9	39	I&D	No	*S. aureus* MS	Levofloxacin + rifampicin	24	Failure	OMR	1	No consolidated
F2010	60 W	Smoker, BMI 22.8	0	I&D	Flap coverage	*Bordetella petrii*	Cotrimoxazole	18	Cure	-	0	Consolidated
F2019	85 W	Dementia, BMI 19.5	565	OMR	No	*S. aureus* MS	Levofloxacin	2	Cure	-	0	Consolidated
F2102	39 M	Smoker, BMI 29.7	4	I&D	No	*E. cloacae*	Ciprofloxacin	24	Cure	-	0	Consolidated
F2103	63 W	DM, BMI 27.8	32	OMR	No	*S. aureus* MS	Clindamycine	12	Cure	-	0	Consolidated
F2204	60 W	Dementia, BMI 22.2	112	OMR	No	*S. aureus* MS	Levofloxacin	20	Cure	-	0	Consolidated
F2206	47 M	BMI 26.4	50	OMR	No	*S. aureus* MS	Amoxicilline	16	Cure	-	0	Consolidated
F2210	41 M	Smoker, BMI 21.4	207	TSR	No	*S. aureus* MS + *P. aeruginosa*	Levofloxacin + rifampicin	43	Failure	TSR	2	No consolidated
F2212	45 W	Pneumopathy, BMI 29.2	26	I&D	No	*S. aureus* MS	Levofloxacin + rifampicin	6	Cure	-	0	Consolidated

BMI: body mass index. DM: diabetes mellitus. I&D: irrigation and debridement. M: man. MS: methicillin-susceptible. OMR: osteosynthesis material removal. PJI: prosthetic joint infection. SAT: suppressive antimicrobial treatment. TSR: two-stage revision. W: woman. * Main antimicrobial therapy is resumed. Early empirical and targeted treatment have been omitted. ** Functional results at last consultation classified as follows: 0—walking without help; 1—needing one crutch; 2—needing two crutches; and 3—in a wheelchair.

**Table 3:** Management and outcome of patients with transfibular ankle prosthetic joint infection.

Case Number	Prosthesis	Days from Implant to Infection	PJI Surgical Therapy	Additional Techniques	Microbiological Aetiology	Antibiotics *	Follow-up/Time to Failure (Months)	Outcome	Salvage Therapy	Functional Outcome **	Radiology at Last Follow-Up
1501	Primary	11	I&D + OMR	Flap coverage	*E. cloacae*	Ciprofloxacin	87	Cure	-	0	Consolidated
1540	Revision	40	I&D	Arthrocentesis	*S. aureus* MS	Levofloxacin + rifampicin	84	Cure	-	0	Consolidated
3002	Primary	49	I&D	Flap coverage + DA	*E. cloacae*	Ciprofloxacin	10	Cure	-	0	Consolidated
1929	Primary	36	I&D	Flap coverage and VAC	*S. aureus* MS *+* *E. cloacae*	Moxifloxacin	19	Failure	TSR (spacer)	0	Consolidated
2010	Primary	82	I&D + OMR	VAC	*S. epidermidis +* *E. faecalis*	Cotrimoxazole + rifampicin	6	Failure	I&D with arthroscopy + SAT	0	Consolidated
1819	Primary	77	I&D + OMR	No	*S. aureus* MS	Levofloxacin + rifampicin	2	Failure	TSR (arthrodesis)	2	Consolidated
3001	Primary	15	I&D	Flap coverage + DA	*S. aureus* MS *+* *E. cloacae* + *K. oxytoca*	Moxifloxacin	7	Failure	I&D + OMR	NA	Consolidated

DA: dissection of anterior compartment. I&D: irrigation and debridement. MS: methicillin-susceptible. NA: not available. OMR: osteosynthesis material removal. PJI: prosthetic joint infection. SAT: suppressive antimicrobial treatment. TSR: two-stage revision. VAC: vacuum therapy. * Main antimicrobial therapy is resumed. Early empirical and targeted treatment have been omitted. ** Functional results at last consultation classified as follows: 0—walking without help; 1—needing one crutch; 2—needing two crutches; and 3—in a wheelchair.

## Data Availability

Data are unavailable due to privacy and ethical restrictions.

## Supplementary Materials

The following supporting information can be downloaded at: https://www.mdpi.com/article/10.3390/antibiotics14030215/s1, Table S1: STROBE list checklist.

## Abbreviations

The following abbreviations are used in this manuscript:

MDPI	Multidisciplinary Digital Publishing Institute
DOAJ	Directory of open access journals
TLA	Three letter acronym
LD	Linear dichroism
